# Deafferentation-Induced Plasticity of Visual Callosal Connections: Predicting Critical Periods and Analyzing Cortical Abnormalities Using Diffusion Tensor Imaging

**DOI:** 10.1155/2012/250196

**Published:** 2012-11-08

**Authors:** Jaime F. Olavarria, Andrew S. Bock, Lindsey A. Leigland, Christopher D. Kroenke

**Affiliations:** ^1^Department of Psychology, Behavioral Neuroscience Program, University of Washington, Box 351525, Seattle, WA 98195-1525, USA; ^2^Advanced Imaging Research Center and Department of Behavioral Neuroscience, Oregon Health and Science University, Mail Code L457, 3181 SW Sam Jackson Park Road, Portland, OR 97239, USA; ^3^Division of Neuroscience, Oregon National Primate Research Center, Oregon Health and Science University, Beaverton, OR 97006, USA

## Abstract

Callosal connections form elaborate patterns that bear close association with striate and extrastriate visual areas. Although it is known that retinal input is required for normal callosal development, there is little information regarding the period during which the retina is critically needed and whether this period correlates with the same developmental stage across species. Here we review the timing of this critical period, identified in rodents and ferrets by the effects that timed enucleations have on mature callosal connections, and compare it to other developmental milestones in these species. Subsequently, we compare these events to diffusion tensor imaging (DTI) measurements of water diffusion anisotropy within developing cerebral cortex. We observed that the relationship between the timing of the critical period and the DTI-characterized developmental trajectory is strikingly similar in rodents and ferrets, which opens the possibility of using cortical DTI trajectories for predicting the critical period in species, such as humans, in which this period likely occurs prenatally. Last, we discuss the potential of utilizing DTI to distinguish normal from abnormal cerebral cortical development, both within the context of aberrant connectivity induced by early retinal deafferentation, and more generally as a potential tool for detecting abnormalities associated with neurodevelopmental disorders.

## 1. Introduction

The establishment of organized patterns of corticocortical connections in sensory systems depends on mechanisms regulating many temporal and spatial aspects of pathway development, including the timing of axon arrival and invasion of gray matter, arborization of axon terminals and dendrites, radial and tangential distribution of neuronal elements, and topographical organization of intrinsic and extrinsic projections. In the visual system, the role that the eyes have on the development of central visual pathways has been studied in anophthalmic animals as well as through various experimental manipulations of visual input, including the removal of retinal afferents through enucleation, dark rearing and visual deprivation following eyelid suture. These previous studies show that that abnormal visual input induces anomalies in both interhemispheric (e.g., reviewed in [1–4]) and intrahemispheric (see references in [5]) pathways. However, these studies also show that visual corticocortical pathways nevertheless develop under the influence of abnormal visual input or in the absence of retinal input, and that, although highly anomalous, they often resemble their normal counterparts in a number of ways. Together, these observations indicate that development of corticocortical circuits depends on the timely interaction between central and peripheral mechanisms. Compared with other approaches for manipulating visual input, enucleation has the advantage that it can be performed long before the eyes open, which facilitates the study of the role of retinal input before the onset of visual experience. In many studies of the effect of enucleation on the development of corticocortical projections, the interhemispheric connection through the corpus callosum has often been the system of choice because the overall distribution of callosal connections in one hemisphere can be readily revealed following multiple injections of anatomical tracers into the opposite hemisphere. Moreover, the distribution of callosal cells and axonal terminations form distinct patterns that extend over broad cortical regions and are consistent among individuals of the same species.

 At what developmental stage is input from the retina critically needed for normal development of corticocortical connections? And, does this critical period correlate with the same stage of brain development in different species? Here we address these questions focusing on the influences the eyes exert on the development of interhemispheric callosal connections in visual cortex and on the size of visual areas. Additionally, we review some recent work in which diffusion tensor imaging (DTI) has been used to directly characterize neuron morphology in developing cerebral cortical gray matter. This approach has potential for predicting the critical period for the effect of retinal deafferentation on the patterns of visual callosal connections in different species, including humans, and for detecting and monitoring abnormal morphological development of the cerebral cortex.

## 2. Retinal Input Is Required during a Brief Neonatal Critical Period in Rodents

**Figure 1:**
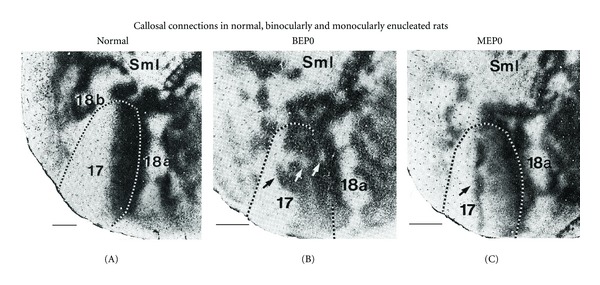
Effect of binocular or monocular enucleation at birth on the pattern of rat visual callosal connections. Callosal patterns in the right hemisphere were revealed following multiple intracortical injections of HRP into the left hemisphere. Images were taken from tangential sections cut through supragranular layers of the flattened cerebral cortex. Dark areas show the distribution of labeled callosal cells and axon terminations. Segmented lines indicate the border of area 17 determined from adjacent sections stained to reveal myeloarchitectonic patterns. Lateral is right, posterior is down. (A) Callosal pattern in normally reared adult rats. Callosal cells and terminations form homogeneously labeled bands at the 17/18a border and at the lateral border of area 18a, and several narrow bands of callosal connections bridge the width of area 18a at several rostrocaudal levels. (B) Binocular enucleation increases the relative width of the callosal band at the 17/18a border and causes the appearance of discrete regions of reduced labeling within the 17/18a callosal band (white arrows) and several densely labeled tongue-like regions that extend medially from this band well into area 17 (black arrow). (C) In rats monocularly enucleated at birth, the most prominent anomaly develops in the hemisphere ipsilateral to the remaining eye, where an anomalous, dense band of callosal connections runs rostrocaudally through the center of area 17 (arrow). Periodic fluctuations in the density of labeling along the length of this extra band give it a beaded appearance. SmI = Somatosensory cortex. Scale bars = 1.0 mm. Adapted from Olavarria et al. [6].

Studies in anophthalmic rats and mice [19–21] have shown that the mature distributions of both intrahemispheric striate-extrastriate and interhemispheric visual callosal connections are abnormal in these animals, indicating that the eyes play an important role in the specification of corticocortical pathways. To examine in more detail the role that the eyes have in the development of visual callosal pathways, Olavarria et al. [6] analyzed the effect of binocular or monocular enucleation performed at postnatal day 0 (P0, within 24 h of birth) on the overall callosal pattern. The effects of binocular (BEP0) and monocular (MEP0) enucleation at birth in the rat are illustrated in Figure 1. In these experiments, the overall callosal patterns in one hemisphere were demonstrated following multiple intracortical injections of the anatomical tracer horseradish peroxidase (HRP) in the other hemisphere. This tracer is transported both anterogradely and retrogradely. Areas containing dense accumulations of callosal cells and axon terminations appear black, and the segmented lines indicate the border of area 17 (striate cortex, primary visual cortex, V1). Figure 1(A) illustrates that in normal rats, callosal cells and terminations form homogeneously labeled bands at the 17/18a border and at the lateral border of area 18a. In addition, several narrow bands of callosal connections bridge the width of area 18a at several rostrocaudal levels. Figure 1(B) shows that binocular enucleation at birth increases the relative width of the callosal band at the 17/18a border and causes the appearance of discrete regions of reduced labeling within the 17/18a callosal band (white arrows in Figure 1(B)) and several densely labeled tongue-like regions that extend medially from this band well into area 17 (black arrow in Figure 1(B)). Moreover, these anomalous features are highly variable across animals [6]. In rats monocularly enucleated at birth, the most prominent anomaly develops in the hemisphere ipsilateral to the remaining eye, where an abnormal, dense band of callosal connections runs rostrocaudally through the center of area 17 (arrow in Figure 1(C)). Periodic fluctuations in the density of labeling along the length of this extra band give it a beaded appearance. The results from experiments using HRP, showing that enucleation induces abnormalities in both the distribution of cell bodies as well as axon terminations, are in agreement with experiments using tracers that are primarily transported either anterogradely or retrogradely [5, 22]. Possible mechanisms leading to distinctly different anomalies of the callosal pattern in binocularly versus monocularly enucleated rodents have been discussed previously [6, 23]. 

 To establish the age at which eye removal ceases to alter the normal course of callosal development, Olavarria et al. [6] delayed the onset of blindness. These experiments showed that development of normal visual callosal patterns requires retinal input during a brief time window extending from postnatal day 4 (P4) to P6 [5, 6]. Indeed, even though the callosal pathway is very immature at P6 [24], removal of the eyes at this age or later does not prevent the development of normal callosal patterns. In contrast, removal of the eyes at P4 or earlier results in patterns that are strikingly abnormal in both their overall distributions [6], as well as in the topographic arrangement of point-to-point callosal linkages [5]. Moreover, virtually the same abnormalities observed in animals enucleated at P4 are also present in animals enucleated at birth, or even in anophthalmic rats [20]. The fact that enucleations at P4 produce results equivalent to enucleations prior to P4 indicates that the eyes do not exert a significant influence on the development of corticocortical connections prior to P4. Therefore, the critical period during which retinal input specifies the overall distribution and point-to-point topography of visual callosal connections occurs in the range from P4 to P6 in rats. A critical period extending from P4 to P6 has also been described for the visual callosal connections in the mouse [5], and a recent study in the rat showed that intrahemispheric striate-extrastriate projections also become immune to the effect of enucleation by P6 [25]. The mechanisms by which retinal input specifies the patterns of corticocortical connections during this critical period are not known at present, but they may involve activity-dependent, as well as chemical cues [5, 6, 25].

 The term critical period used here refers to a well-defined developmental stage during which the presence of a specific factor (in this case the retinae) is critically required (hence critical period) for development to proceed normally [26]. These critical periods typically occur at early stages of development and have been described in various systems at different levels of the neuroaxis (see, e.g., [27]). It is important to note that these critical periods differ from periods occurring later in life, during which functional and anatomical changes reflect changes in sensory experience that do not necessarily require end organ damage [26]. For example, in rat visual cortex, vision deprivation experiments have demonstrated a period of ocular dominance plasticity that extends approximately from P18 to well into the second month of life [28]. Unfortunately, as pointed out by Erzurumlu and Killackey [26], the term critical period (as well as the term sensitive period) has been used to refer to multiple temporally and mechanistically distinct phases of development (see, e.g., [29]).

## 3. Need for Information about the Critical Period in Other Species

The previous studies in rodents described above show that lack of retinal input during a brief critical period induces permanent alterations in the overall distribution and topography of visual intra- and interhemispheric corticocortical pathways. Thus, the period identified by delaying enucleation represents a unique developmental stage during which retinal input is critically needed for specifying the normal layout and topography of corticocortical connections. Mapping out this critical period is therefore important for identifying the retinally driven mechanisms that operate at this developmental stage, and for investigating how they lay down the blueprints for normal maps of corticocortical connectivity. However, while early enucleation has been shown to affect the development of corticocortical connections in several other species, including monkeys [30, 31], cats [9, 32], hamsters [23, 33, 34], and opossum [35], there is little or no information about the beginning and end of the critical period in these or other species. This may be in part because in many species, the critical period for the effect of retinal input on the distribution and topography of corticocortical connections likely ends during gestation, making the experimental mapping of the critical period difficult.

Due to the lack of comparative data about the timing of the critical period, a question that remains unanswered is whether the critical period correlates with the same stage of central visual development in different species. This question is important because the finding that the critical period correlates with a specific developmental stage across species would suggest that the eyes guide the development of corticocortical connections through a similar mechanism in all species. Moreover, this finding would facilitate predicting the critical period in species in which this period occurs prenatally, as is likely the case in humans, as well as interpreting previous observations in other species that seem to deviate from expected outcomes. For instance, the effects of bilateral enucleation at birth on callosal connections are significantly less severe in cats [9, 32, 36] than in rats [6, 37] and ferrets [8]. Since cats are born at more advanced stages of development compared to rodents and ferrets, these observations raise that possibility that the critical period during which retinal influences specify the pattern of callosal connections ends prenatally in the cat [8]. 

## 4. Effects of Neonatal Retinal Deafferentation on the Callosal Pattern in the Ferret, and Comparison with the Effects in the Cat

**Figure 2:**
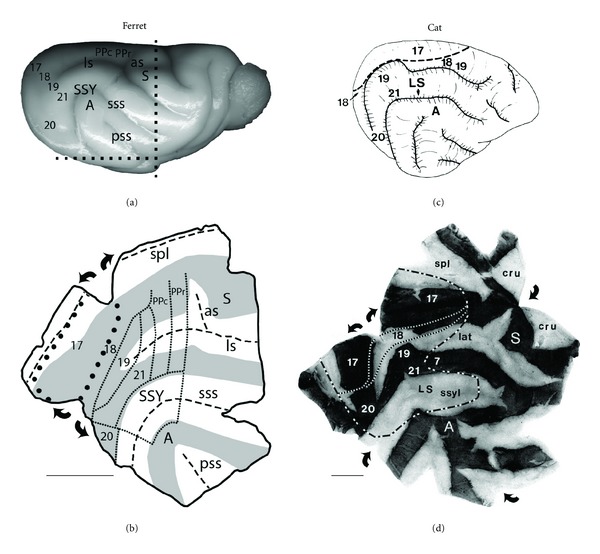
Comparing the arrangements of gyri, sulci, and visual areas in the unfolded and flattened cortex of ferrets and cats. Posterior is left, dorsal is up. (a) Intact right hemisphere of ferret brain showing the location of gyri, sulci and various cortical areas. Dashed lines indicate cuts made to separate the posterior block that was unfolded and flattened. (b) Diagram showing an unfolded and flattened ferret cortex. Curved arrows indicate further cuts made to fully flatten the cortex. Dashed lines indicate fundi of sulci, gray areas indicate the surface of gyri, large dotted line indicates the outline of V1, and lines made of small dots indicate the borders of other visual areas. These landmarks and lines are useful for correlating features of the callosal pattern to the patterns of gyri, sulci, and visual areas (see Figures 3(a), 3(b), and 3(c)). A: auditory cortex; as: ansate sulcus; ls: lateral sulcus; PPc: posterior parietal caudal; PPr: posterior parietal rostral; pss: pseudosylvian sulcus; S: somatosensory cortex; spl: splenial sulcus; sss: suprasylvian sulcus; SSY: suprasylvian visual area. (c) Schematic diagram of intact right hemisphere of cat brain showing location of various visual areas. Dashed line indicates lateral border of area 17. (d) Diagram showing an unfolded and flattened cat cortex. Curved arrows indicate cuts made to fully flatten the cortex. Dark and light areas correspond to gyri and sulci, respectively. Fine dashed lines indicate the lateral borders of areas 17 and 18. The heavy dashed line indicates the approximate location of several visual areas, including areas 18, 19, 20, 21, and suprasylvian areas. These lines are also useful for relating features of the callosal patterns to visual areas (see Figures 3(d), 3(e), and 3(f)). Unfolding and flattening the cortex of ferrets and cats were performed as described previously [7]. A: auditory cortex; S: somatosensory cortex; spl: splenial; lat: lateral; cru: cruciate; ssyl: suprasylvian. Scale bars = 1.0 cm. Adapted from Bock et al. [8]; Olavarria and Van Sluyters [9].

To address the lack of information about the critical period in carnivores, we studied the effect of neonatal enucleation on the callosal pattern in the ferret [8] taking advantage of the fact that these animals are born at relatively early stages of central nervous system (CNS) development [38]. We characterized the abnormalities induced by neonatal enucleation on the distribution and number of callosal cells in striate and extrastriate cortex and explored the period during which the presence of the eyes is required for the normal development of the visual callosal pattern in this species. The callosal patterns in normal adult ferrets were compared to those in adult ferrets that had been enucleated at P7 or P20, ages that approximately correspond to a period spanning from P2.2 to P8 in the rat [14, 15]. Following multiple intracortical injections of horseradish peroxidase (HRP) into one hemisphere, patterns of callosal connections were studied in tangential sections cut through striate and extrastriate cortex of the unfolded and flattened contralateral hemisphere [8]. The locations of visual areas described in previous physiological studies were estimated from the relation of these areas to gyral and sulcal landmarks [39, 40]. In Figure 2(a), the location of visual areas, as well as somatosensory and auditory areas, are indicated in the intact ferret brain, while in Figure 2(b), the borders of visual (fine dotted lines) areas have been drawn across gyri (in gray) and sulci (in white) on the unfolded and flattened cortical mantle. Corresponding information for the cat is shown on Figures 2(c) and 2(d) for comparison [9].

**Figure 3:**
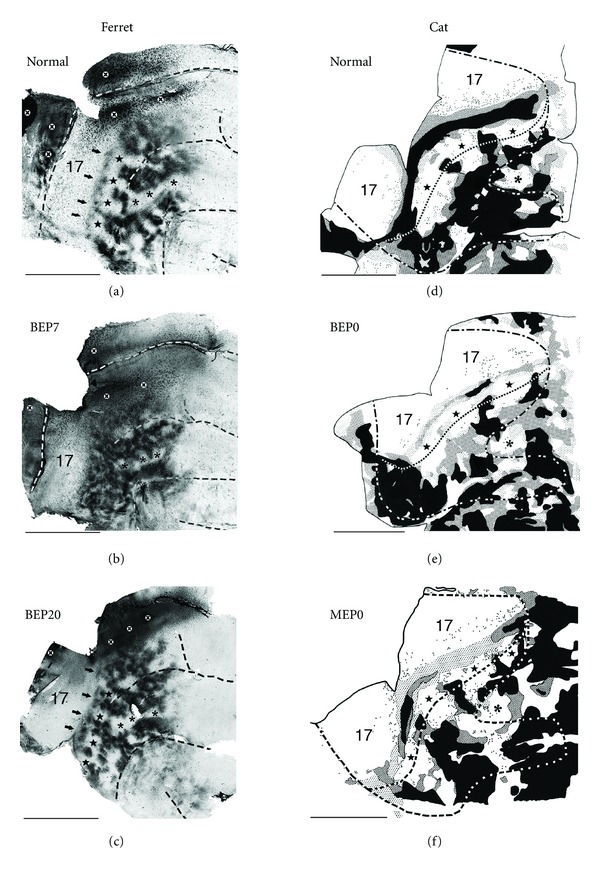
Effects of enucleation on the visual callosal patterns in ferrets and cats. Callosal patterns were revealed on the right hemisphere following multiple intracortical injections of HRP into the left hemisphere. Callosal patterns were reconstructed from several tangential sections cut through the unfolded and flattened cortex (see Figure 2). Posterior is left, dorsal is up. (a, b, c) Visual callosal pattern in normal (a), bilaterally enucleated at postnatal day 7 (BEP7) (b), and bilaterally enucleated at postnatal day 20 (BEP20) (c) ferrets. Dark areas correspond to callosal cell bodies and axonal terminations labeled with HRP reaction product. Dashed lines indicate fundi of sulci. Black stars on the lateral gyrus in (a) and (c) indicate acallosal regions common to all control and BEP20 ferrets. Asterisks on the suprasylvian gyrus indicate acallosal regions common to all control, BEP7, and BEP20 ferrets; black arrows indicate string of callosal patches located in area 18 in control (a) and BEP20 (c) ferrets. Black X's within white circles in (a, b, c) indicate regions of artifactual labeling. (d, e, f) Visual callosal patterns in normal (d), bilaterally enucleated at birth (BEP0) (e), and monocularly enucleated at birth (MEP0) (f) cats. Black regions indicate areas in which the density of labeled cells was highest while dark and light stippling represent areas of decreasing density. Dots represent single labeled cells. The fine dashed line indicates the approximate location of the lateral border of area 18. The lateral border of area 17 (represented by a fine dashed line in Figure 2(d)) is not explicitly marked in (d), (e), and (f) because it approximately runs along the middle of the 17/18 callosal band (the continuous band of callosal connections separating areas 17 and 18). The heavy dashed line corresponds to the outline of visual areas in Figure 2(d). Black stars on the lateral sulcus in (d), (e), and (f) indicate acallosal regions observed in areas 18 and 19 in all control and enucleated cats, and the asterisks on the suprasylvian gyrus indicate acallosal regions common to all control and enucleated cats. Scale bars = 1.0 cm. Adapted from Bock et al. [8]; Olavarria and Van Sluyters [9].

The overall callosal pattern in normal ferrets (Figure 3(a)) bears some resemblance to that reconstructed in cats from tangential sections cut through the unfolded and flattened cortex (Figure 3(d)) [9], particularly in those callosal features associated with visual areas identified as areas 17,18, 19, and 20 in both species [40–42]. In ferrets (Figure 3(a); see also [39]), as in cats (Figure 3(d)), two parallel bands of callosal connections can be identified, one straddling the border between areas 17 and 18, and the other located at the lateral border of area 19. These bands and a series of bridge-like bands or patches extending between them at different anteroposterior locations separate several acallosal areas (marked with black stars in Figures 3(a), and 3(d)). The callosal bands correspond with representations of central visual fields in both species [40, 43], while the acallosal islands correspond to areas representing peripheral visual fields in areas 18 and 19 in both cats [44] and ferrets [40]. The callosal-free zone we observed on the suprasylvian gyrus in all control ferrets (marked with black asterisks in Figure 3(a)) may be homologous with an area devoid of callosal connections on the suprasylvian gyrus of normal cats [9]. In the ferret, this area seems to be shared by several visual areas mapped recently, including middle portions of areas 21, and lateral portions of areas PPc and PPr [45]. Further laterally, in the suprasylvian sulcus, several patches of callosal labeling are observed in both normal ferrets and cats, which appear to correspond to areas of central visual field representation within the lateral suprasylvian visual areas of the cat [46], and within visual area SSY of the ferret [47, 48].

## 5. Enucleation at P7, but Not at P20, Induces Highly Anomalous Patterns of Callosal Connections in Ferrets

Enucleation at P7 induces marked anomalies in the distribution of callosal connections in the ferret [8]. In area 17, the distribution of labeled callosal cells is relatively sparse as in control ferrets (Figure 3(b)). However, observations at higher magnification show that, unlike in control ferrets, callosal neurons are not restricted to regions near the anterior border of area 17, but are also observed in broader regions of this area. It is possible that the more widespread distribution of callosal cells in area 17 of BEP7 ferrets derives from an exuberant distribution present at early stages of normal development, as reported in other species (e.g., [6]). Testing this possibility will require performing tracer injection experiments in area 17 of immature ferrets. In extrastriate cortex, the callosal pattern in BEP7 ferrets consists of patches of labeled cells and axon terminations that are smaller and more numerous than in controls, and they often occupy regions that are relatively free of callosal labeling in control animals (cf. Figures 3(a) and 3(b)). Several features that are readily seen in all control ferrets are therefore either absent or difficult to recognize in BEP7 ferrets, including the string of callosal patches located in area 18 close to the area 17 border (indicated by arrows in Figure 3(a)), as well as the acallosal areas that are consistently present in areas 18 and 19 of control ferrets (marked with black stars in Figure 3(a)). However, other features of the callosal pattern in control ferrets are recognizable in BEP7 ferrets, such as the acallosal area on the suprasylvian gyrus (marked with black asterisks in Figures 3(a), and 3(b)), although this acallosal area appears somewhat smaller in BEP7 ferrets. Based on previous physiological subdivisions of ferret visual cortex [40, 49], these data indicate that regions showing abnormal patterns of callosal connectivity encompassed many visual areas, including areas 18, 19, and 21.

 In sharp contrast to observations in BEP7 ferrets, by P20 the development of callosal connections is no longer susceptible to disruption of visual input by binocular enucleation. Indeed, the callosal patterns in ferrets enucleated at P20 are virtually indistinguishable from those in control ferrets (cf. Figures 3(a) and 3(c)). These results indicate that the development of the overall pattern of callosal connections depends critically on retinal influences within the time range extending from P7 to P20. Supporting the impression that callosal connections in BEP7 ferrets tend to fill in spaces that are callosal free in controls, the percentage of extrastriate visual cortex occupied by callosal connections is significantly increased in BEP7 ferrets compared with controls. In contrast, no significant differences in this percentage are observed in BEP20 ferrets compared with controls, supporting the observation that the callosal pattern in BEP20 ferrets (Figure 3(c)) closely resembles that in controls (Figure 3(a)). Moreover, within the extrastriate visual region analyzed, the average number of cells per histological section is not significantly different among control, BEP7, and BEP20 ferrets [8].

Interestingly, in cats, the effects of enucleation at birth (BEP0, Figure 3(e)) resemble those observed in BEP20 ferrets in that enucleation fails to induce significant changes in the overall pattern of callosal connections in both striate and extrastriate cortices (cf. Figures 3(d) and 3(e), [9]). These results suggest that the critical period for the effect of enucleation on callosal connections ends before birth in the cat (see below). However, unlike BEP20 in ferrets, BE0 in cats induces a significant reduction in the number of callosal cells in striate end extrastriate visual cortex [9, 36], suggesting, as discussed below, that visual deprivation following enucleation has different effects in these two species.

## 6. Is the Critical Period for the Specification of Callosal Connections Prenatal in the Cat?

The effects of enucleation at P7 on the callosal pattern in ferrets (Figure 3(b); [8]) and prior to P4 in rats [6, 37] appear much more severe than those observed in adult cats bilaterally enucleated at birth [9, 32, 36]. For instance, enucleation at P0 in cats primarily reduces the number of callosal cells without significantly changing the overall callosal pattern at the 17/18 border and extrastriate cortex (cf. Figures 3(d) and 3(e)), although the reduction in the number of callosal neurons is less dramatic in extrastriate cortex than at the 17/18 callosal zone [9, 36]. The differences between BEP0 cats and BEP7 ferrets are particularly dramatic in extrastriate cortex. While in BEP7 ferrets callosal connections fill in most regions that are free of connections in normal animals (cf. Figures 3(a) and 3(b)), features of the normal pattern in extrastriate cortex are readily recognized in BEP0 cats (cf. Figures 3(d) and 3(e)). It is possible that bilateral enucleation has a smaller effect in cats because the critical period may have ended by the time of birth. Support for this idea comes from the fact that, at birth, development of corticocortical connections in the cat resembles the developmental stages observed in rats and ferrets by the end of the critical period. For example, by P6 in rats [22, 50] and P20 in ferrets [51], corticocortical axons of simple morphology have begun invading upper layers of the cortex just as supragranular layers differentiate from the cortical plate [38, 52, 53]. At birth, cats are at an approximately similar developmental stage [54–56], suggesting that by P0 the critical period during which retinal influences specify the pattern of callosal connections has already ended in this species. A similar conclusion derives from using the Translating Time [14, 15] model to translate to the cat developmental stages from rats and ferrets. Thus, P6 in rats translates to 61.9 gestational days in cats (gestation in cats lasts 65 days), and P20 in ferrets translates to 66.8 gestational days, or 1.8 postnatal days, in cats. Additionally, Issa et al. ([57], see their Figure 7) equate P0 in the cat with P23 in the ferret, based on the near coincidence in the timing of several developmental milestones when age is expressed in terms of days post conception, rather than days following birth. Consistent with these conclusions, monocular enucleation at birth in the cat fails to induce obvious anomalies in the distribution of callosal connections in striate and extrastriate cortex in the hemisphere ipsilateral to the remaining eye (cf. Figures 3(d) and 3(f)) (Olavarria, unpublished results). This is in marked contrast with studies in rats [6] and hamsters [23], in which monocular enucleation prior to P4 causes the development of an extra, anomalous band of callosal connections in the middle regions of area 17 ipsilateral to the remaining eye (Figure 1(c)). It will be interesting to investigate whether prenatal enucleations in cats induce the development of callosal patterns as anomalous as those we observed in BEP7 ferrets. These considerations support the idea that the critical period correlates with the same stage of central visual development in different species. If correct, this relationship would indicate that it is possible to predict the critical period in species, such as humans, in which this period occurs prenatally, provided sufficient information about the developmental profile for such species is available. As discussed below, studies using noninvasive MRI techniques have revealed that brain development over a range that overlaps the critical period for the specification of normal callosal connections is characterized by consistent changes in water diffusion anisotropy in the cerebral cortex of a variety of species, including humans, which makes the prediction of developmental landmarks such as critical periods possible.

While the idea that the critical period in cats has ended by P0 explains why the layout of the callosal pattern was largely undisturbed in cats enucleated at P0, it does not explain why enucleation after the critical period for callosal pattern formation leads to a significant loss of callosal cells, especially at the 17/18 border. This loss in cell number is best explained as additional evidence that, in the cat, early visual experience is required to maintain the integrity of neuronal circuits and response properties that are attained during normal development independently from vision [58–61]. Surprisingly, in adult ferrets enucleated at P20 [8] and adult rats and mice enucleated at P6 [5, 6], the callosal pattern closely resembles that in normal adult animals in both number and distribution of callosal neurons, indicating that, unlike cats, visual experience is not required for the maintenance of a normal complement of callosal neurons in these species. Additional comparative studies will be required for understanding why different species depend on early visual experience to a different extent for the maintenance of normal complements of corticocortical neurons.

## 7. A Longer Critical Period for the Effect of Enucleation on the Size of Cortical Visual Areas

Several reports have provided evidence that early enucleation leads to a reduction in the surface area of striate cortex [6, 30, 31, 35, 62, 63]. Studies of the effect of fetal enucleation in macaques have concluded that the reduction in the size of striate cortex is accompanied by a partial respecification of the neighboring cortex into an additional, hybrid visual cortex, called area X [62, 63], so that the overall surface area of the neocortex remains unchanged [31]. Recently, Reillo et al. [64] showed in the ferret that bilateral enucleation at P1 induces a 35–40% reduction in the size of striate cortex. However, unlike earlier findings in enucleated macaques, these authors found that the surface area of the cerebral cortex was also reduced, which led them to propose that enucleation at P1 does not lead to the respecification of striate cortex or enlargement of neighboring areas. Bock et al. [8] measured the cortical surface area of striate and extrastriate cortex using MRI data obtained from a number of ferret brains that were also used for analyzing the callosal pattern in the same study. Enucleation at P7 was found to cause a significant reduction in the size of striate cortex relative to controls. Compared with the reduction reported by Reillo et al. [64] in BEP1 ferrets, the reduction in striate cortex was on average smaller in BEP7 ferrets (25.6%). Although enucleation at P20 does not induce obvious anomalies in the callosal pattern, it still produces a significant reduction of the size of striate cortex. However, the reduction in size was smaller (18.3%) than that observed in BEP7 ferrets, suggesting that the effect of enucleation is greater for earlier enucleations, as previously reported for the macaque monkey [31, 63]. Enucleation at P7 and P20 reduced the size of extrastriate cortex by about 15% on average. This effect is smaller than that observed in striate cortex, perhaps because the effect of enucleation may be primarily restricted to only parts of the total area we measured in extrastriate cortex, a possibility that is worth exploring in future studies. In agreement with Reillo et al. [64], these data from striate and extrastriate cortex suggest that the reduction in striate cortex does not result in the respecification of striate cortex or the enlargement of extrastriate areas as reported previously in the monkey [31, 62, 63]. Additional studies will be required to explain why visual cortex of macaques and ferrets respond differently to early binocular enucleation. In agreement with findings in the ferret, Laing et al. [25] reported that, on average, the size of area 17 in BEP0, BEP4 and BEP6 rats was 61%, 71.4%, and 83.9% of the size of area 17 in controls rats, respectively. These results showed that the effect of bilateral enucleation on the size of striate cortex was greater for earlier enucleations, and that although smaller, the reduction in the size of area 17 was still significant by P6, when enucleation no longer induces anomalies in the callosal pattern. However, as discussed in more detail below, the size of area 18a in the rat was not significantly affected by enucleation at any age studied. Preliminary results from our laboratory suggest that the critical period for the effect of enucleation on the size of striate cortex in the rat ends by the end of the second postnatal week, at about the time of eye opening (A. Andelin and J. Olavarria, unpublished observations). In cats, unpublished measurements based on the anatomical data from three normal and three BEP0 cats presented by Olavarria and Van Sluyters [9] indicate that enucleation at birth causes a 39.8% reduction in the size of striate cortex (on average, a reduction from 339.46 mm^2^ to 204.36 mm^2^), and a 34.8% reduction in the size of extrastriate cortex (on average, a reduction from 510.75 mm^2^ to 332.70 mm^2^) as defined in this previous study (the region containing areas 18,19, 20, 21, and LS in Figure 2(d)). When one considers that the critical period for the effect of enucleation on callosal connections in the cat appears to end prenatally (see discussion above), the effects of bilateral enucleation at P0 on the size of visual areas in cats seem disproportionally large compared with the average reduction in the size of area 17 caused by enucleations performed at P6 in rats (16.1%) and P20 in ferrets (18.3%). This intriguing observation adds to the finding described above that enucleation at birth in cats produces a significant reduction in the number of callosal cells without altering the overall pattern of callosal connections [9, 36]. These observations suggest that in cats, stabilization of normal numbers of callosal neurons as well as the size of visual areas may depend on visual experience after eye opening to a greater extent than in rats and ferrets.

The findings in ferret and rat that enucleation still reduces the size of visual areas even if performed when it no longer affects the development of callosal patterns suggests that retinal influences continue to regulate the size of visual cortex after the patterns of visual corticocortical connections have become specified. These results further suggest that retinal influences regulate several aspects of cortical development through mechanisms that do not necessarily operate during the same time window. Moreover, they indicate that the pattern of visual corticocortical connections can develop without obvious abnormalities even if striate cortex does not reach its normal size. It is interesting to note that the finding that area 17, but not area 18a, is reduced in enucleated rats [25] contrasts with the observation that enucleation reduces the size of both striate and extrastriate cortex in ferrets and cats. Projections from the dLGN have been implicated in the regulation of the size of visual areas (see [8], for references relevant to this issue). In this context, these differences are probably related to the fact that projections from the dLGN are largely restricted to area 17 in the rat [65–67], while in the ferret and cat, in addition to area 17, direct retinogeniculate projections innervate area 18 and possibly other extrastriate visual areas [39, 68–72]. Previous studies in the monkey have shown that early enucleation reduces both the size of the dLGN as well as the number of fibers ascending from this nucleus [31, 62]. Subsequent studies have linked the effect of enucleation on the size of striate cortex to the modulatory influences that thalamic afferents, by means of diffusible factors, exert over early stages of corticogenesis at the level of the ventricular zone [73]. Reillo et al. [64] illustrated that enucleation at P1 induces a noticeable reduction in the size of the dLGN in the ferret, raising the possibility that the reduction in the size of visual cortex observed in BEP20 ferrets and BEP6 rats reflects a protracted effect of enucleation on the size of the dLGN. Further studies are needed to determine the effects of enucleation at different ages on the sizes of both the dLGN and striate cortex, and to compare these effects to corresponding data from monkeys [31].

While enucleation at P7 in ferrets reduces the size of extrastriate cortex and induces the development of abnormal patterns of callosal connections, it does not cause significant changes in the number of labeled callosal cells in this cortical region compared to control ferrets. Since this cortical region is smaller in BEP7 ferrets, this observation is consistent with the finding that the percentage of extrastriate visual cortex occupied by callosal connections is significantly larger in BEP7 than in control ferrets [8]. It will be of interest to perform stereological studies to investigate whether this result applies only to callosal cells, or whether the total number of neurons is preserved in extrastriate cortex of early enucleates, as it appears to be the case in striate cortex of BEP1 ferrets [64]. It is tempting to speculate that the marked changes observed in the callosal pattern of BEP7 ferrets may be due, at least in part, to rearrangements imposed by the need to accommodate approximately the same number of callosal neurons in a significantly smaller cortical area. Along this line of thought and in light of the differential effect of bilateral enucleation on the sizes of areas 17 and 18a in the rat [25], it is interesting to note that abnormalities in the callosal pattern are generally more severe in area 17 than in area 18a in this rodent (cf. Figures 1(A) and 1(B)) [6]. 

## 8. Predicting Critical Periods Using Diffusion Tensor Imaging

Studies in ferrets [8], rats, and mice [5] indicate that retinal influences are necessary for the development of normal callosal patterns during a brief, well defined critical period early in development. Moreover, comparison of these results with developmental data available in these species suggests that the critical period correlates with similar stages of central visual development across species (see below). However, at present it is difficult to accurately predict the critical period in many species with prolonged gestation, such as monkeys and humans, because detailed information about prenatal brain development obtained with standard anatomical techniques is lacking in these species. In what follows we explore the possibility of predicting the critical period for a certain species by comparing information about its brain development obtained with non-invasive magnetic resonance imaging (MRI) techniques with corresponding MRI data from another species with a known critical period.

Diffusion tensor imaging (DTI), a recently developed magnetic resonance imaging (MRI) technique, measures parameters of water diffusion anisotropy that depend on the shape and orientation of cellular elements, such as somas, axons, and dendrites [74]. For instance, water diffusion is less restricted along the length of axons and dendrites than in an orthogonal direction because of the impediment imposed by cell membranes [75]. Due to these properties, DTI shows great potential as a non-invasive technique for studying the development of cellular architecture and connectivity under normal as well as pathological conditions. Moreover, because DTI measurements are influenced by developmental changes in all cellular, axonal, and neuropil (axons, dendrites, and associated extracellular space) components in brain tissue, this technique can provide a picture of the developmental trajectory of the brain that is more comprehensively representative, albeit less specific, than information obtained with histological methods traditionally used for studying CNS development at the systems level. Information on developmental changes in DTI measurements is currently available for many species including rats, ferrets, and humans (see below), making it possible to predict the critical period for a certain species based on the association between the critical period and developmental DTI measurements determined in another species.

**Figure 4:**
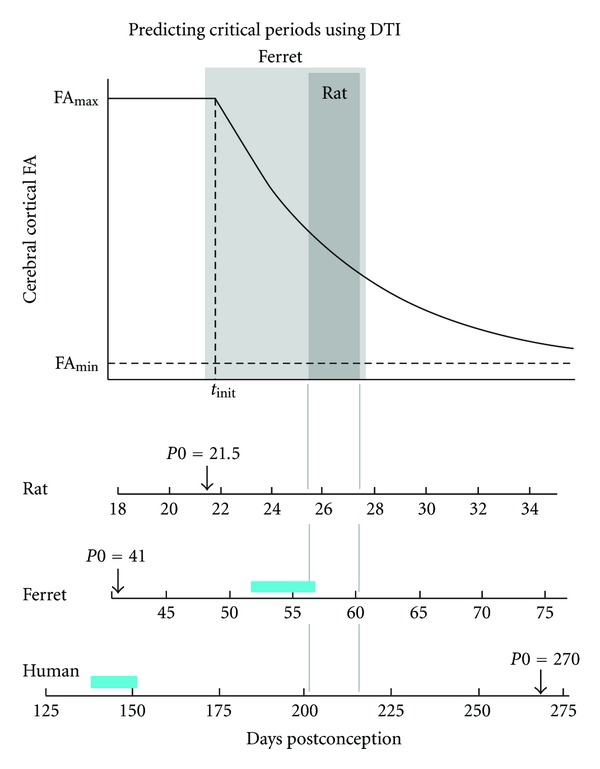
Water diffusion anisotropy (FA) within the developing cerebral cortex versus postconceptional age. The upper trace is a plot of the dependence of cerebral cortical FA on age, as given by (1) (see text). Below, abscissas for the plot are given in terms of postconceptional ages in days, using *t*_init_ and *τ* values obtained by analyzing rat data reported by Huang et al. [10], ferret data from Kroenke et al. [11], and human data from McKinstry et al. [12], following procedures described in [13] (see text for details). The dark gray shaded area represents the lower and upper age bounds of the critical period for callosal connectivity using data available for the rat [5, 6] (the rat critical period, P4–P6, corresponds to 25.5–27.5 days post conception). The light gray shaded area represents the ages explored in the ferret [8] (P7 and P20, which correspond to 48 and 61 days post conception, resp.). The duration in days of gestation up to birth (P0) is indicated for each species (arrows). Gray lines indicate time range corresponding to the critical period in rats (P4–P6), which predicts critical periods for ferret (P15–P19; PC56–PC60) and human (PC201–PC217) according to the cortical FA trajectory. Blue bars indicate time ranges translated from the rat critical period (P4–P6) to ferret (P11–15.5; PC52–PC56.5) and human (PC138–PC150), according to the Translating Time model developed by Finlay, and coworkers [14–17].

The DTI technique is most frequently used to study properties of white matter [74]. In the developing cerebral cortex, water diffusion is influenced by cellular structures that are different from those that influence water diffusion in white matter. In developing white matter, water diffusion anisotropy increases with maturation of axon tracts, in association with several neural developmental events including the formation of mature myelin [74, 76–78]. However, in the developing cerebral cortex, it is the reduction in water diffusion anisotropy that is related to morphological differentiation. At stages prior to the onset of myelin formation, immediately after pyramidal neurons of the isocortex migrate from germinal zones to the cortical plate, the neuropil consisting primarily of neuronal and glial processes and the associated extracellular space begins to differentiate [79]. Dendrites and axons start to develop as simple elongated structures, oriented perpendicular to the pial surface, an arrangement that induces anisotropy in water diffusion because it selectively restricts water diffusion in directions parallel to the pial surface [12, 76]. As dendrites and axons arborize to form interconnected, functional neural circuits [80], diffusion within cortex becomes increasingly restricted in all directions, causing anisotropy of water diffusion to become progressively smaller, although still measurable, in the mature brain [81]. A commonly used parameter to quantify diffusion anisotropy is fractional anisotropy (FA, [82]) which ranges from 0 (isotropic diffusion) to 1 (extremely anisotropic diffusion). The idea that the reduction in FA associated with cortical development arises from morphological differentiation of the neuropil [12] is supported by the fact that the age-related decreases in cerebral cortical FA coincide with developmental changes in neuropil morphology [11, 83]. In order to quantify cortical FA changes in absolute terms of CNS development, it has been proposed that FA decreases exponentially from a maximal value (FA_max⁡_) to the minimal value (FA_min⁡_) observed at maturity [11]. In a simplified form of the expression proposed in [11], appropriate for assessing average FA throughout the isocortex, and in which the terms reflecting regional patterns in cortical FA have been ignored [13], FA can be considered to depend on age

(1) $$\begin{array}{c} \text{FA}\left(\text{age}\right)=\{\begin{array}{cc} {\text{FA}}_{\max}, & \text{if}\;\text{age}<t_{\text{init}},\\ \left({\text{FA}}_{\max}-{\text{FA}}_{\min}\right)\exp\left(-\frac{\text{age}}{\tau }\right)+{\text{FA}}_{\min}, & \text{if}\;\text{age}\geq \;t_{\text{init}}, \end{array} \end{array}$$

as illustrated in Figure 4. In this expression, the two parameters *t*_init_ and *τ* relate FA to the trajectory of cortical development. The parameter *t*_init_ reflects the time in which pyramidal neurons have completed neurogenesis and migration to the cortical plate. The exponential decay time constant, *τ*, reflects the rate of morphological development of the neuropil. Importantly, for many species including humans, cortical FA changes take place prenatally. Therefore, to facilitate interspecies comparisons, the ages represented in Figure 4 are expressed as days post conception even when they fall after birth.

The progressive loss of cortical diffusion anisotropy with age has been quantified for rat [10, 84, 85], mouse [86, 87], ferret [11, 88], baboon [83], and human [12, 89–91]. To assess the consistency of developmental stage dependent FA changes between species, Leigland and Kroenke [13] analyzed published cortical FA data using the above expression, in which the value of *t*_init_ for each species corresponds to the time period immediately following the genesis and subsequent migration of pyramidal neurons from ventricular/subventricular zones to the cortical plate [11]. These authors found a high degree of correlation between *τ* and rates of neuroanatomical development quantified through large-scale comparative meta analyses of CNS development (see below) [15–17].

## 9. The Critical Period for the Effect of Enucleation on Callosal Connections Coincides with Similar Stages of Cortical FA Decay in Rodents and Ferrets

When the critical periods in rodents and ferrets are correlated with the developmental trajectories revealed by cortical FA, it becomes apparent that the critical period occurs over a remarkably similar developmental time span. In Figure 4, the top graph illustrating how cerebral cortical FA decays with development represents available data from rat, ferret, and human. In order to fit the same curve, the data from each of these groups were transformed by shifting each curve so that the *t*_init_ values coincided and by scaling each species' age by a factor proportional to 1/*τ*. Analysis of the data presented by Huang et al. [10] in the rat yields values of 22 days and 5 days, respectively, for *t*_init_ and *τ* (in which *t*_init_ is expressed as days post conception (PC); *t*_init_ is postnatal day 0.5 for the rat). Thus, for the rat, the critical period for interhemispheric connectivity specification corresponds to times in which cortical FA ranges from 0.50 (on day P4; PC 25.5) to 0.33 (on day P6; PC 27.5) of the difference between FA_max⁡_ and FA_min⁡_ (area shaded dark gray in Figure 4). By comparison, the P7–P20 (PC48–PC61) age range investigated in enucleated ferrets (light gray area in Figure 4) begins just prior to *t*_init_ and extends until FA is 0.33 of the difference between FA_max⁡_ and FA_min⁡_. The values used for ferret *t*_init_ and *τ* were 49 days post conception (which corresponds to P8) and 10.7 days, respectively [11]. It should be noted that the critical period has been determined with greater precision in rodent species than in ferrets. It is therefore likely that future studies will reveal that the ferret critical period extends through a shorter time range. For instance, based on the relationship between the critical period and FA curve in the rat, we predict that the critical period for the ferret would extend approximately from P15 to P20 (PC56 to PC61, between the gray lines in Figure 4). For humans, the FA curve is based on data from McKinstry et al., 2002 [12], in which *t*_init_ is 173 days post conception and *τ* is 39.8 days. Assuming that the critical period in humans relates to the FA decay curve as in rats, we predict that the critical period for cortical connectivity in humans extends from about 201 days (28.7 weeks) to 217 days (31 weeks) of gestation (between the gray lines in Figure 4).

An alternative procedure for correlating developmental events across animal species was developed by Finlay and coworkers [14–17]. Using published data identifying the timing of specific developmental events obtained by standard anatomical methods from many species, these authors derived a time model, called Translating Time (http://translatingtime.org/public/translate). This model is based on three factors (a species score, an event score, and an interaction term). The species score reflects the rate of development of a particular species. The event score reflects the timing of specific neural developmental events. The interaction term reflects a slower rate of cerebral cortical development in primates, relative to other structures in the CNS. In addition to predicting the ages of many species at specific neurodevelopmental events, this model can also be used to translate the age of one species to that of another. An in-depth comparison of interspecies DTI studies to results obtained using the translating time model is given in [13]. Using this model the critical period determined in the rat and mouse (P4–P6; PC25.5–PC27.5) translates to a period extending from about 138 days (19.7 weeks) to 150 days (21.4 weeks) of gestation in humans. As illustrated in Figure 4, the critical period for humans predicted this way (blue bar) is significantly earlier than the one predicted using the relationship between the critical period and FA decay curve established with data from the rat (between the gray lines in Figure 4). In contrast, predictions for the ferret critical period based on either cortical FA data (between gray lines) or the translating time model (from P11 to P15.5, corresponding to PC52 to PC56.5, blue bar in Figure 4) are relatively close to one another. It should be noted that the ranges for both predictions for the ferret lie within the P7–P20 (PC48–PC61) range examined experimentally in this species [8], and that further studies will be required to test these predictions in this species.

Our prediction of the critical period in humans based on the FA decay curve is consistent with available developmental data. In rodents and ferrets, fibers from the thalamus penetrate layer 4 of the cerebral cortex before the onset of the critical period. In rodents, thalamic fibers project to layer IV in visual cortex as early as P3 [92], while in the ferret fibers of thalamic origin can be observed in cortical layer IV by the end of the first postnatal week [93]. By P6 in rats [22, 50] and P20 in ferrets [51], corticocortical axons of simple morphology begin invading upper cortical layers just as supragranular layers differentiate from the cortical plate [38, 52, 53]. These data suggest that the end of the critical period in rodents and ferrets correlates with the arrival of axon terminals into supragranular layers and the beginning of arbor branching, which may contribute to the decrease of cortical FA.

In humans, Burkhalter et al. [94] described the development of cortical architecture and local connections within visual cortex in postmortem brains using the anatomical tracer carbocyanine dye DiI and bisbenzimide counterstaining of tissue sections. Following DiI injections into the thalamic radiation below primary visual cortex of a 26-week-old human fetus (182 gestation days), these authors observed that presumed thalamocortical afferents had entered all layers of the cortical plate forming a well-defined bundle of fibers oriented perpendicular to the pia. Moreover, in histological sections through visual cortex of a 29-week-old human fetus (203 gestation days) they showed that layers 2/3 were clearly differentiated from layers 4 and lower layers, a pattern they described as “emerging cortical layers”. These data are consistent with our prediction based on FA cortical changes that the critical period in humans takes place between 201 and 217 days of gestation (Figure 4). If accurate, our estimate of the critical period in humans will be valuable for differentiating between visual deficiencies secondary to early prenatal damage of the visual pathway from those due to pathologies of postnatal onset.

## 10. Using Diffusion Tensor Imaging to Detect and Monitor Cortical Abnormalities Induced by Bilateral Neonatal Enucleation in the Ferret

Certain neurodevelopmental disorders are thought to be associated with abnormalities in morphological differentiation of the cerebral cortical neuropil [95]. Therefore, in addition to using cortical FA to characterize the tempo of CNS development, it is of interest to investigate the possibility of also using FA measurements to develop strategies for detecting and monitoring the deleterious effects of pathological insults on cortical development, as well as for assessing the efficacy of therapeutic interventions initiated at early stages of development while the brain is still plastic. Previous studies have associated blindness with abnormalities of the cerebral cortical neuropil. For instance, Golgi studies of animals that have been dark-reared [96, 97], stripe-reared [98], or binocularly-enucleated [99, 100] have documented effects on several aspects of dendritic development in the cerebral cortex, including abnormalities in dendritic fields of pyramidal cells, and reductions in the number of dendritic spines. At the cellular level, enucleation increases the length of callosal axon branches and total length of arbors, without major effects on the number of branch tips [101], and reduces the proportion of multiple synaptic boutons in the visual callosal projection [102]. In view of the massive effects that neonatal enucleation in the ferret has on visual callosal connections, Bock et al. [18] decided to use this animal model to explore the potential of DTI techniques for detecting abnormal cortical development induced by enucleation. These authors compared cortical FA between control and BEP7 ferrets using values measured at P31, when neuronal morphological differentiation is still underway.

**Figure 5:**
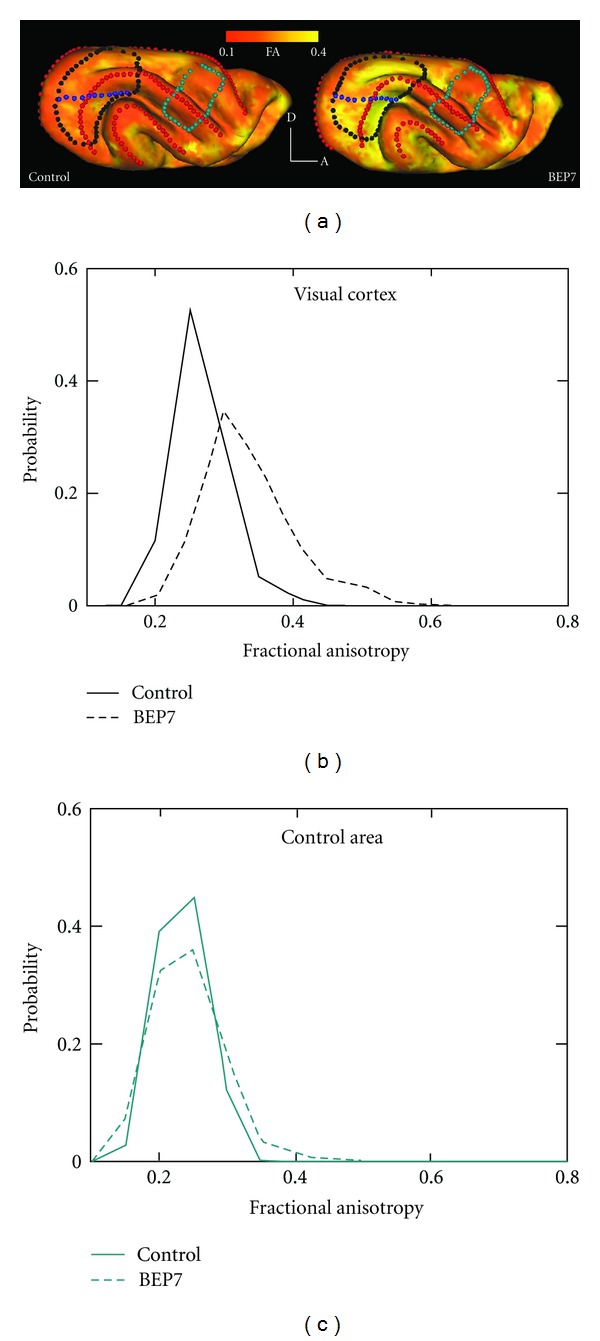
Visual cortical FA is increased in BEP7 ferrets, relative to controls, at P31. (a) Cortical FA is projected onto lateral cortical surface models for one control and one BEP7 ferret, according to the red/yellow color scale (posterior is left, dorsal is up). The BEP7 surface exhibits greater FA (brighter yellow) than the control in visual areas (encircled by black dots) at P31, while no differences in FA are apparent in a control, nonvisual area located more rostrally (encircled by blue-green dots). Blue dots indicate the approximate location of the horizontal meridian representation. Red dots indicate crowns of gyri. (b, c) Histograms represent data from either two BEP7 or two control ferrets. Comparison of these histograms illustrates that differences in cortical FA are observed in visual areas (b), but not in the nonvisual control area (c). Adapted from Bock et al. [18].

Due to the widespread distribution of callosal connections in the brain, the overall cortical area affected by enucleation can be readily estimated by determining which regions contain abnormal callosal patterns. In turn, this greatly facilitates the identification of areas to be analyzed with DTI methods (Figure 5(a)). Data from one BEP7 animal and one control are illustrated in Figure 5(a), in which cortical FA has been projected onto lateral views of ferret cerebral cortical surface models. This figure illustrates that the BEP7 surface exhibits greater FA (brighter yellow) than the control in visual areas (encircled by black dots) at P31, while no differences in FA are apparent in a control, non-visual area located more rostrally (encircled by blue-green dots). Figures 5(b) and 5(c) compare histograms from two BEP7 and two control ferrets, and illustrate that differences in cortical FA are observed in visual areas (Figure 5(b)), but not in the non-visual control area (Figure 5(c)). These results show that cortical visual areas exhibiting differences in callosal connectivity at adulthood were spatially correlated with regions exhibiting altered cortical FA at P31. In agreement with these results in ferrets, a preliminary comparison performed at P6 between normal and P0-enucleated rats revealed that anisotropy within deep layers of primary visual cortex is increased in the enucleated rats compared with normal animals [103]. These results provide further evidence that binocular enucleation perturbs the normal development of visual cortex, and supports the notion that DTI is capable of detecting changes in connectivity associated with binocular enucleation at early stages of brain development. It is important to note that, in addition to the abnormalities induced on callosal connections, enucleation at P7 in the ferret likely affects several other visual connection systems, including thalamocortical and ipsilateral, intrahemispheric corticocortical projections [104–108]. It is therefore possible that the effect on FA within cerebral cortical gray matter observed over much of visual cortex of BEP7 ferrets reflects the effect of enucleation on multiple pathways that either terminate or originate in visual cortex.

**Figure 6:**
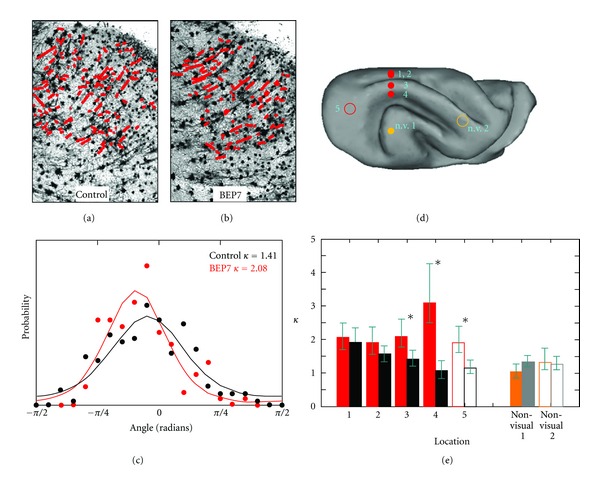
Increased cerebral cortical diffusion anisotropy in the developing visual cortex accompanies reduction in the neuronal process orientation distribution width. (a, b) Golgi-stained visual cortex tissue visualized at 10x magnification from a P31 control (a) and BEP7 (b) ferret. Line segments (red) representing neuronal processes throughout a region of the cerebral cortex (corresponding to location 3 in (d)) are overlaid on (a), and on an image obtained from the same region of a BEP7 visual cortex at P31 in (b). The polar angle for each line segment was determined, and in (c), histograms representing the distribution of polar angles are shown for the control (black data points) and BEP7 (red data points) Golgi fields. (c) Solid lines represent the results of approximating the data points as a Von Mises distribution. The distribution observed for the BEP7 field is narrower, and hence characterized by a larger concentration parameter *κ*, than for the control. (d) Location of 5 other regions analyzed in control and BEP7 ferrets. Filled circles indicate fields obtained from coronal sections, and open circles from axial sections. Filled and open orange circles indicate the locations of nonvisual areas (n.v.). (e) Concentration parameters for the 5 other region of the visual cortex, obtained from pairs of coronal sections (solid bars in (e)) and axial sections (open bars in (e)). For all 5 visual regions, *κ* is larger for BEP7 animals (red solid and open bars) than for controls (black solid and open bars). For visual locations indicated with asterisks, 95% confidence intervals for BEP7 and control regions do not overlap [18]. Filled and open orange bars in (e) represent data analyzed for nonvisual regions of BEP7 cortices, while filled and open gray bars in e represent regions of nonvisual cortex in control cortices. Significant differences for nonvisual areas are not observed. Adapted from Bock et al. [18].

In order to directly examine the cellular-level determinants of the differences between control and BEP7 animals observed by DTI, a procedure was developed to quantitatively characterize orientation distributions of neuronal processes in Golgi-stained cerebral cortical tissue (Figure 6) [18]. For sets of visual and non-visual cortical locations, Golgi-stained neurons were compared between control and BEP7 animals. Figures 6(a) and 6(b) show that, in the P31 cortex, radially-oriented apical dendrites appear as dominant structures. To quantify the distributions of Golgi-stained processes in BEP7 and control animals, the images were skeletonized, and the resulting stained segments were approximated as lines (red overlays, Figures 6(a) and 6(b) [18]). Figure 6(c) shows that the distribution of line segment polar angles for the BEP7 animal is narrower than it is for the control Golgi field. When fitted to a Von Mises distribution, it was found that the BEP7 field is characterized by a higher concentration parameter, *κ* = 2.08, than for the control field, in which *κ* = 1.41. Figure 6(e) shows 7 such comparisons between BEP7 and control cerebral cortices, at locations indicated in Figure 6(d). In visual areas, the concentration parameters were higher for BEP7 than control ferrets, consistent with a more uniform distribution of apical dendrites for BEP7 ferrets compared to controls, whereas such differences were not observed in non-visual areas. These data suggest that the more coherent orientation of apical dendrites observed in BEP7 ferrets accounts, at least in part, for increases in cortical FA measured in visual cortex of these animals.

## 11. Comparison with Other Studies and Future Directions for the Use of Diffusion Tensor Imaging to Characterize Cerebral Cortical Neuropil Differentiation

One question that arises is whether abnormal development of cellular elements not detectable by Golgi staining, such as radial glial cells, underlies the cortical FA differences measured in enucleated animals. For example, one study presenting both DTI and immunohistochemical data on cerebral cortical microarchitecture in the neonatal rat brain [84] concluded that FA perturbations associated with hypoxic ischemic injury were related to the quantity of neuronal and glial fibers oriented approximately parallel to apical dendrites. In normal ferrets, radial glial cells differentiate into astrocytes by P21 [109], suggesting that they do not contribute significantly to cortical FA at the age this analysis was performed (P31). Moreover, the possibility that enucleation somehow delays differentiation of radial glia is unlikely because studies in other species have shown that enucleation does not affect the timing of other developmental milestones, such as the formation of topographically organized corticocortical projections [6, 22]. Lastly, the findings reported in the ferret are consistent with a study in the rat [101], which showed that bilateral enucleation increased the length of axon branches and arbors without changing the number of branches, as well as a study in the rat showing that visual deprivation alters dendritic bundle architecture in rat visual cortex [110], and a study in the mouse showing a reduction in the number of spines on apical dendrites of pyramids in bilaterally enucleated animals [99]. Together, these results suggest that DTI is capable of detecting abnormalities induced by bilateral enucleation on the differentiation of axonal and dendritic arbors in visual cortex.

Several recent studies have described approaches for quantitatively comparing light microscopy-based measurements of axon and/or dendrite fiber orientations to DTI results. Overall, the strategy commonly used by these studies is similar to ours [18], namely, diffusion MRI measurements are performed on aldehyde-fixed post mortem tissue, the tissue is subsequently sectioned and stained, and an image analysis procedure is applied to determine the statistical distribution of fiber orientations within a specified region of interest. Leergaard et al. [111] and Choe et al. [112] have conducted analyses in which diffusion MRI data from white matter were compared to 2D fiber orientation distributions measured from myelin-stained tissue. These two studies differed in their analysis of microscopy data. While Leergaard and co-workers manually traced fibers, Choe et al. performed an automated Fourier analysis to generate fiber orientation distributions. Finally, Budde and co-workers [113] used Fourier analyses of images of cerebral cortical tissue following various neuronal and glial staining procedures to reveal a role of gliosis in cortical FA changes in adult cerebral cortex following traumatic brain injury. To address limitations of 2D analysis, we have recently extended the analysis of Golgi-stained tissue in the developing ferret cerebral cortex to 3D by performing confocal microscopy of reflected light [114]. This recent work will likely facilitate future improvements in the precision with which water diffusion MRI measurements of brain cellular morphology can be validated.

The study by Bock et al. [18] provides evidence that neonatal bilateral enucleation induces alterations of neuronal processes that can be detected by DTI at early stages of development, but some issues remain to be addressed in future studies. First, the developmental trajectory of the difference between normal and bilaterally enucleated ferrets must be measured by examining brains at postnatal ages other than P31 in order to identify the developmental stage in which DTI is most sensitive for detecting abnormal morphological development of cerebral cortical neurons. Second, while the study of Bock et al. [18] shows that DTI has sufficient sensitivity to detect enucleation-induced changes in neuronal morphology using postmortem tissue, it is possible that sensitivity differs when the procedure is performed in live animals. Postmortem DTI studies are instrumental in studies that validate DTI data with subsequent histological analyses, but future experiments involving DTI measurements in live ferrets and incorporating rapid image acquisition techniques such as echoplanar imaging will be needed to explore the possibility of extending these finding to living tissue in detail.

## 12. Conclusion

In conclusion, studies in rodents and ferret indicate that normal development of interhemispheric and intrahemispheric corticocortical connections requires retinal input during brief critical periods at early stages of pathway formation. Moreover, these critical periods correlate with similar stages of brain development in these animals, suggesting that the eyes guide the development of corticocortical connections through common mechanisms across species. Based on the observation that the relationship between the timing of the critical period and the DTI-characterized developmental trajectory is strikingly similar in rodents and ferrets, we explored the possibility of using cortical DTI trajectories for predicting the critical period in species, such as humans, in which this period likely occurs prenatally. If accurate, our estimate of the critical period in humans will be useful for differentiating between visual deficiencies secondary to early prenatal damage of the visual pathway from those due to pathologies of postnatal onset. Finally, the MRI findings reviewed above indicate that the effect of neonatal enucleation on the development of callosal and other neuronal systems in the ferret provide an ideal experimental model for investigating the sensitivity of DTI for detecting abnormalities in neuronal architecture. Specifically, the model described is amenable for systematically investigating how patterns of water diffusion anisotropy within the cerebral cortex change during early stages of development. Such studies will be useful for understanding the morphological factors underlying the DTI findings in human studies of developmental disorders of the CNS. By avoiding potential confounds related to *in utero* manipulations or preterm birth, the use of newborn ferrets will greatly facilitate DTI analysis of changes in diffusion anisotropy at stages of brain development that in primate species occur before birth. The studies reviewed here thus provide a strategy for using DTI to identify abnormalities early in brain development, thereby enabling therapeutic intervention before significant reduction of brain plasticity occurs.

## References

[1] Innocenti GM (1991). The development of projections from cerebral cortex. *Progress in Sens Physiology*.

[2] Innocenti GM, Price DJ (2005). Exuberance in the development of cortical networks. *Nature Reviews Neuroscience*.

[3] Olavarria JF (2002). Influence of topography and ocular dominance on the functional organization of callosal connections in the cat striate cortex. *The Cat Primary Visual Cortex*.

[4] Pietrasanta M, Restani L, Caleo M (2012). The corpus callosum and the visual cortex: plasticity is a game for two. *Neural Plasticity*.

[5] Olavarria JF, Hiroi R (2003). Retinal influences specify cortico-cortical maps by postnatal day six in rats and mice. *Journal of Comparative Neurology*.

[6] Olavarria J, Malach R, Van Sluyters RC (1987). Development of visual callosal connections in neonatally enucleated rats. *Journal of Comparative Neurology*.

[7] Olavarria J, Van Sluyters RC (1985). Unfolding and flattening the cortex of gyrencephalic brains. *Journal of Neuroscience Methods*.

[8] Bock AS, Kroenke CD, Taber EN, Olavarria JF (2012). Retinal input influences the size and corticocortical connectivity of visual cortex during postnatal development in the ferret. *The Journal of Comparative Neurology*.

[9] Olavarria JF, Van Sluyters RC (1995). Overall pattern of callosal connections in visual cortex of normal and enucleated cats. *Journal of Comparative Neurology*.

[10] Huang H, Yamamoto A, Hossain MA, Younes L, Mori S (2008). Quantitative cortical mapping of fractional anisotropy in developing rat brains. *Journal of Neuroscience*.

[11] Kroenke CD, Taber EN, Leigland LA, Knutsen AK, Bayly PV (2009). Regional patterns of cerebral cortical differentiation determined by diffusion tensor MRI. *Cerebral Cortex*.

[12] McKinstry RC, Mathur A, Miller JH (2002). Radial organization of developing preterm human cerebral cortex revealed by non-invasive water diffusion anisotropy MRI. *Cerebral Cortex*.

[13] Leigland LA, Kroenke CD (2011). A comparative analysis of cellular morphological differentiation within the cerebral cortex using diffusion tensor imaging. *Neuromethods*.

[14] Clancy B, Darlington RB, Finlay BL (2001). Translating developmental time across mammalian species. *Neuroscience*.

[15] Clancy B, Kersh B, Hyde J, Darlington RB, Anand KJS, Finlay BL (2007). Web-based method for translating neurodevelopment from laboratory species to humans. *Neuroinformatics*.

[16] Finlay BL, Darlington RB (1995). Linked regularities in the development and evolution of mammalian brains. *Science*.

[17] Darlington RB, Dunlop SA, Finlay BL (1999). Neural development in metatherian and eutherian mammals: variation and constraint. *The Journal of Comparative Neurology.*.

[18] Bock AS, Olavarria JF, Leigland LA, Taber EN, Jespersen SN, Kroenke CD (2010). Diffusion tensor imaging detects early cerebral cortex abnormalities in neuronal architecture induced by bilateral neonatal enucleation:An experimental model in the ferret. *Frontiers in Systems Neuroscience*.

[19] Bravo H, Inzunza O (1994). Effect of pre- and postnatal retinal deprivation on the striate-peristriate cortical connections in the rat. *Biological Research*.

[20] Olvarria J, Bravo H, Ruiz G (1988). The pattern of callosal connections in posterior neocortex of congenitally anophthalmic rats. *Anatomy and Embryology*.

[21] Olavarria J, Van Sluyters RC (1984). Callosal connections of the posterior neocortex in normal-eyed, congenitally anophthalmic, and neonatally enucleated mice. *Journal of Comparative Neurology*.

[22] Olavarria JF, Safaeian P (2006). Development of callosal topography in visual cortex of normal and enucleated rats. *Journal of Comparative Neurology*.

[23] O'Brien BJ, Olavarria JF (1995). Anomalous patterns of callosal connections develop in visual cortex of monocularly enucleated hamsters. *Biological Research*.

[24] Olavarria J, Van Sluyters RC (1985). Organization and postnatal development of callosal connections in the visual cortex of the rat. *Journal of Comparative Neurology*.

[25] Laing RJ, Bock AS, Lasiene J, Olavarria JF (2012). Role of retinal input on the development of striate-extrastriate patterns of connections in the rat. *The Journal of Comparative Neurology*.

[26] Erzurumlu RS, Killackey HP (1982). Critical and sensitive periods in neurobiology. *Current Topics in Developmental Biology*.

[27] Belford GR, Killackey HP (1980). The sensitive period in the development of the trigeminal system of the neonatal rat. *Journal of Comparative Neurology*.

[28] Fagiolini M, Pizzorusso T, Berardi N, Domenici L, Maffei L (1994). Functional postnatal development of the rat primary visual cortex and the role of visual experience: dark rearing and monocular deprivation. *Vision Research*.

[29] Crowley JC, Katz LC (2000). Early development of ocular dominance columns. *Science*.

[30] Dehay C, Horsburgh G, Berland M, Killackey H, Kennedy H (1989). Maturation and connectivity of the visual cortex in monkey is altered by prenatal removal of retinal input. *Nature*.

[31] Dehay C, Giroud P, Berland M, Killackey H, Kennedy H (1996). Contribution of thalamic input to the specification of cytoarchitectonic cortical fields in the primate: effects of bilateral enucleation in the fetal monkey on the boundaries, dimensions, and gyrification of striate and extrastriate cortex. *Journal of Comparative Neurology*.

[32] Innocenti GM, Frost DO (1980). The postnatal development of visual callosal connections in the absence of visual experience or of the eyes. *Experimental Brain Research*.

[33] Rhoades RW, Dellacroce DD (1980). Neonatal enucleation induces an asymmetric pattern of visual callosal connections in hamsters. *Brain Research*.

[34] Fish SE, Rhoades RW, Bennett-Clarke CA, Figley B, Mooney RD (1991). Organization, development and enucleation-induced alterations in the visual callosal projection of the hamster: single axon tracing with Phaseolus vulgaris leucoagglutinin and Di-I. *European Journal of Neuroscience*.

[35] Karlen SJ, Krubitzer L (2009). Effects of bilateral enucleation on the size of visual and nonvisual areas of the brain. *Cerebral Cortex*.

[36] Olavarria JF (1995). The effect of visual deprivation on the number of callosal cells in the cat is less pronounced in extrastriate cortex than in the 17/18 border region. *Neuroscience Letters*.

[37] Cusick CG, Lund RD (1982). Modification of visual callosal projections in rats. *Journal of Comparative Neurology*.

[38] Jackson CA, Peduzzi J, Hickey TL (1989). Visual cortex development in the ferret. I. Genesis and migration of visual cortex neurons. *Journal of Neuroscience*.

[39] Innocenti GM, Manger PR, Masiello I, Colin I, Tettoni L (2002). Architecture and callosal connections of visual areas 17, 18, 19 and 21 in the ferret (Mustela putorius). *Cerebral Cortex*.

[40] Manger PR, Kiper D, Masiello I (2002). The representation of the visual field in three extrastriate areas of the ferret (Mustela putorius) and the relationship of retinotopy and field boundaries to caliosal connectivity. *Cerebral Cortex*.

[41] Tusa RJ, Palmer LA, Rosenquist AC (1978). The retinotopic organization of area 17 (striate cortex) in the cat. *Journal of Comparative Neurology*.

[42] Tusa RJ, Rosenquist AC, Palmer LA (1979). Retinotopic organization of areas 18 and 19 in the cat. *Journal of Comparative Neurology*.

[43] Payne BR (1990). Representation of the ipsilateral visual field in the transition zone between areas 17 and 18 of the cat's cerebral cortex. *Visual Neuroscience*.

[44] Sanides D, Albus K (1980). The distribution of interhemispheric projections in area 18 of the cat: coincidence with discontinuities of the representation of the visual field in the second visual area (V2). *Experimental Brain Research*.

[45] Manger PR, Masiello I, Innocenti GM (2002). Areal organization of the posterior parietal cortex of the ferret (Mustela putorius). *Cerebral Cortex*.

[46] Palmer LA, Rosenquist AC, Tusa RJ (1978). The retinotopic organization of lateral suprasylvian visual areas in the cat. *Journal of Comparative Neurology*.

[47] Cantone G, Xiao J, Levitt JB (2006). Retinotopic organization of ferret suprasylvian cortex. *Visual Neuroscience*.

[48] Manger PR, Engler G, Moll CKE, Engel AK (2008). Location, architecture, and retinotopy of the anteromedial lateral suprasylvian visual area (AMLS) of the ferret (Mustela putorius). *Visual Neuroscience*.

[49] Manger PR, Nakamura H, Valentiniene S, Innocenti GM (2004). Visual areas in the lateral temporal cortex of the ferret (Mustela putorius). *Cerebral Cortex*.

[50] Ruthazer ES, Bachleda AR, Olavarria JF (2010). Role of interstitial branching in the development of visual corticocortical connections: a time-lapse and fixed-tissue analysis. *Journal of Comparative Neurology*.

[51] Juliano SL, Palmer SL, Sonty RV, Noctor S, Hill GF (1996). Development of local connections in ferret somatosensory cortex. *The Journal of Comparative Neurology*.

[52] Ignacio MPD, Kimm EJ, Kageyama GH, Yu J, Robertson RT (1995). Postnatal migration of neurons and formation of laminae in rat cerebral cortex. *Anatomy and Embryology*.

[53] Herrmann K (1996). Differential distribution of AMPA receptors and glutamate during pre- and postnatal development in the visual cortex of ferrets. *The Journal of Comparative Neurology*.

[54] Shatz CJ, Luskin MB (1986). The relationship between the geniculocortical afferents and their cortical target cells during development of the cat's primary visual cortex. *Journal of Neuroscience*.

[55] Price DJ, Zumbroich TJ (1989). Postnatal development of corticortical efferents from area 17 in the cat's visual cortex. *Journal of Neuroscience*.

[56] Aggoun-Zouaoui D (1996). Growth of callosal terminal arbors in primary visual areas of the cat. *European Journal of Neuroscience*.

[57] Issa NP, Trachtenberg JT, Chapman B, Zahs KR, Stryker MP (1999). The critical period for ocular dominance plasticity in the Ferret's visual cortex. *Journal of Neuroscience*.

[58] Fregnac Y, Imbert M (1978). Early development of visual cortical cells in normal and dark-reared kittens: relationship between orientation selectivity and ocular dominance. *Journal of Physiology*.

[59] Innocenti GM, Frost DO, Illes J (1985). Maturation of visual callosal connections in visually deprived kittens: a challenging critical period. *Journal of Neuroscience*.

[60] Crair MC, Gillespie DC, Stryker MP (1998). The role of visual experience in the development of columns in cat visual cortex. *Science*.

[61] Zufferey PD, Jin F, Nakamura H, Tettoni L, Innocenti GM (1999). The role of pattern vision in the development of cortico-cortical connections. *European Journal of Neuroscience*.

[62] Rakic P (1988). Specification of cerebral cortical areas. *Science*.

[63] Rakic P, Suner I, Williams RW (1991). A novel cytoarchitectonic area induced experimentally within the primate visual cortex. *Proceedings of the National Academy of Sciences of the United States of America*.

[64] Reillo I, De Juan Romero C, García-Cabezas MA, Borrell V (2011). A Role for intermediate radial glia in the tangential expansion of the mammalian cerebral cortex. *Cerebral Cortex*.

[65] Olavarria J (1979). A horseradish peroxidase study of the projections from the latero-posterior nucleus to three lateral peristriate areas in the rat. *Brain Research*.

[66] Ribak CE, Peters A (1975). An autoradiographic study of the projections from the lateral geniculate body of the rat. *Brain Research*.

[67] Schober W, Winkelmann E (1977). The geniculo cortical projections in albino rats. *Journal fur Hirnforschung*.

[68] Friedlander MJ, Martin KAC (1989). Development of Y-axon innervation of cortical area 18 in the cat. *Journal of Physiology*.

[69] Humphrey AL, Sur M, Uhlrich DJ, Sherman SM (1985). Termination patterns of individual X- and Y-cell axons in the visual cortex of the cat: projections to area 18, to the 17/18 border region, and to both areas 17 and 18. *Journal of Comparative Neurology*.

[70] Redies C, Diksic M, Riml H (1990). Functional organization in the ferret visual cortex: a double-label 2-deoxyglucose study. *Journal of Neuroscience*.

[71] Ruthazer ES, Baker GE, Stryker MP (1999). Development and organization of ocular dominance bands in primary visual cortex of the sable ferret. *Journal of Comparative Neurology*.

[72] White LE, Bosking WH, Williams SM, Fitzpatrick D (1999). Maps of central visual space in Ferret V1 and V2 lack matching inputs from the two eyes. *Journal of Neuroscience*.

[73] Dehay C, Savatier P, Cortay V, Kennedy H (2001). Cell-cycle kinetics of neocortical precursors are influenced by embryonic thalamic axons. *Journal of Neuroscience*.

[74] Mori S, Zhang J (2006). Principles of diffusion tensor imaging and its applications to basic neuroscience research. *Neuron*.

[75] Beaulieu C (2002). The basis of anisotropic water diffusion in the nervous system—a technical review. *NMR in Biomedicine*.

[76] Neil JJ, Shiran SI, McKinstry RC (1998). Normal brain in human newborns: apparent diffusion coefficient and diffusion anisotropy measured by using diffusion tensor MR imaging. *Radiology*.

[77] Wimberger DM, Roberts TP, Barkovich AJ, Prayer LM, Moseley ME, Kucharczyk J (1995). Identification of ‘premyelination’ by diffusion-weighted mri. *Journal of Computer Assisted Tomography*.

[78] Lebel C, Walker L, Leemans A, Phillips L, Beaulieu C (2008). Microstructural maturation of the human brain from childhood to adulthood. *NeuroImage*.

[79] Rakic P (1995). A small step for the cell, a giant leap for mankind: a hypothesis of neocortical expansion during evolution. *Trends in Neurosciences*.

[80] Conel JL (1939). *The Postnatal Development of the Human Cerebral Cortex*.

[81] McNab JA, Jbabdi S, Deoni SCL, Douaud G, Behrens TEJ, Miller KL (2009). High resolution diffusion-weighted imaging in fixed human brain using diffusion-weighted steady state free precession. *NeuroImage*.

[82] Basser PJ, Pierpaoli C (1996). Microstructural and physiological features of tissues elucidated by quantitative-diffusion-tensor MRI. *Journal of Magnetic Resonance B*.

[83] Kroenke CD, Van Essen DC, Inder TE, Rees S, Bretthorst GL, Neil JJ (2007). Microstructural changes of the baboon cerebral cortex during gestational development reflected in magnetic resonance imaging diffusion anisotropy. *Journal of Neuroscience*.

[84] Sizonenko SV, Camm EJ, Garbow JR (2007). Developmental changes and injury induced disruption of the radial organization of the cortex in the immature rat brain revealed by in vivo diffusion tensor MRI. *Cerebral Cortex*.

[85] Bockhorst KH, Narayana PA, Liu R (2008). Early postnatal development of rat brain: in vivo diffusion tensor imaging. *Journal of Neuroscience Research*.

[86] Baloch S, Verma R, Huang H (2009). Quantification of brain maturation and growth patterns in C57BL/6J mice via computational neuroanatomy of diffusion tensor images. *Cerebral Cortex*.

[87] Larvaron P, Boespflug-Tanguy O, Renou JP, Bonny JM (2007). In vivo analysis of the post-natal development of normal mouse brain by DTI. *NMR in Biomedicine*.

[88] Barnette AR, Neil JJ, Kroenke CD (2009). Characterization of brain development in the ferret via MRI. *Pediatric Research*.

[89] Huang H, Xue R, Zhang J (2009). Anatomical characterization of human fetal brain development with diffusion tensor magnetic resonance imaging. *Journal of Neuroscience*.

[90] Gupta RK, Hasan KM, Trivedi R (2005). Diffusion tensor imaging of the developing human cerebrum. *Journal of Neuroscience Research*.

[91] DeIpolyi AR, Mukherjee P, Gill K (2005). Comparing microstructural and macrostructural development of the cerebral cortex in premature newborns: diffusion tensor imaging versus cortical gyration. *NeuroImage*.

[92] Kageyama GH, Robertson RT (1993). Development of geniculocortical projections to visual cortex in rat: evidence for early ingrowth and synaptogenesis. *Journal of Comparative Neurology*.

[93] Herrmann K, Antonini A, Shatz CJ (1994). Ultrastructural evidence for synaptic interactions between thalamocortical axons and subplate neurons. *European Journal of Neuroscience*.

[94] Burkhalter A, Bernardo KL, Charles V (1993). Development of local circuits in human visual cortex. *Journal of Neuroscience*.

[95] Nitkin RM (2000). Dendritic mechanisms in brain function and developmental disabilities. *Cerebral Cortex*.

[96] Borges S, Berry M (1978). The effects of dark rearing on the development of the visual cortex of the rat. *Journal of Comparative Neurology*.

[97] Coleman PD, Riesen AH (1968). Evironmental effects on cortical dendritic fields. I. Rearing in the dark. *Journal of Anatomy*.

[98] Tieman SB, Hirsch HVB (1982). Exposure to lines of only one orientation modifies dendritic morphology of cells in the visual cortex of the cat. *Journal of Comparative Neurology*.

[99] Heumann D, Rabinowicz T (1982). Postnatal development of the visual cortex of the mouse after enucleation at birth. *Experimental Brain Research*.

[100] Ryugo R, Ryugo DK, Killackey HP (1975). Differential effect of enucleation on two populations of layer V pyramidal cells. *Brain Research*.

[101] Olavarría JF, Laing R, Hiroi R, Lasiene J (2008). Topography and axon arbor architecture in the visual callosal pathway: effects of deafferentation and blockade of *N*-methyl-*D*-aspartate receptors. *Biological Research*.

[102] Sorensen SA, Jones TA, Olavarria JF (2003). Neonatal enucleation reduces the proportion of callosal boutons forming multiple synaptic contacts in rat striate cortex. *Neuroscience Letters*.

[103] Kroenke CD, Bock AS, Taber EN, Olavarria JF (2008). Use of diffusion tensor magnetic resonance imaging to monitor anatomical differentiation of the cerebral cortex in normal and enucleated rats. *Society for Neuroscience*.

[104] Berman NEJ (1991). Alterations of visual cortical connections in cats following early removal of retinal input. *Developmental Brain Research*.

[105] Ankaoua D, Malach R (1993). Evidence for plasticity of intrinsic horizontal connections in area 17 of the rat. *Israel Journal of Medical Sciences*.

[106] Toldi J, Fehér O, Wolff JR (1996). Neuronal plasticity induced by neonatal monocular (and binocular) enucleation. *Progress in Neurobiology*.

[107] Ruthazer ES, Stryker MP (1996). The role of activity in the development of long-range horizontal connections in area 17 of the ferret. *Journal of Neuroscience*.

[108] Karlen SJ, Kahn DM, Krubitzer L (2006). Early blindness results in abnormal corticocortical and thalamocortical connections. *Neuroscience*.

[109] Voigt T (1989). Development of glial cells in the cerebral wall of ferrets: direct tracing of their transformation from radial glia into astrocytes. *Journal of Comparative Neurology*.

[110] Gabbott PL, Stewart MG (2012). Visual deprivation alters dendritic bundle architecture in layer 4 of rat visual cortex. *Neuroscience*.

[111] Leergaard TB, White NS, de Crespigny A (2010). Quantitative histological validation of diffusion MRI fiber orientation distributions in the rat brain. *PLoS ONE*.

[112] Choe AS, Stepniewska I, Colvin DC, Ding Z, Anderson AW (2012). Validation of diffusion tensor MRI in the central nervous system using light microscopy: quantitative comparison of fiber properties. *NMR in Biomedicine*.

[113] Budde MD, Janes L, Gold E, Turtzo LC, Frank JA (2011). The contribution of gliosis to diffusion tensor anisotropy and tractography following traumatic brain injury: validation in the rat using Fourier analysis of stained tissue sections. *Brain*.

[114] Jespersen SN, Leigland LA, Cornea A, Kroenke CD (2012). Determination of axonal and dendritic orientation distributions within the developing cerebral cortex by diffusion tensor imaging. *IEEE Transactions on Medical Imaging*.

